# Brd4 regulates the expression of essential autophagy genes and Keap1 in AML cells

**DOI:** 10.18632/oncotarget.24432

**Published:** 2018-02-07

**Authors:** Min Huang, Li Zhu, Jacqueline S. Garcia, Michael X. Li, Andrew J. Gentles, Beverly S. Mitchell

**Affiliations:** ^1^ Department of Medicine, Stanford Cancer Institute, Stanford University, Stanford, California, USA; ^2^ Department of Pathology, Stanford University School of Medicine, Stanford, California, USA; ^3^ Department of Medicine, Dana-Farber Cancer Institute, Boston, Massachusetts, USA; ^4^ Department of Electrical Engineering and Computer Science, College of Engineering, Oregon State University, Corvallis, Oregon, USA; ^5^ Department of Medicine, Biomedical Informatics Research, Stanford University, Stanford, California, USA

**Keywords:** Brd4, autophagy, AML, Keap1

## Abstract

We have recently reported that activation of Brd4 is associated with the presence of autophagy in NPMc+ and MLL AML cells. In order to determine the mechanisms underlying this relationship, we have examined the role of Brd4 in regulating the expression of several genes that are central to the process of autophagy. We found that Brd4 binds to the promoters of ATG 3, 7 and CEBPβ, and expression of these genes is markedly reduced by inhibitors of Brd4, as well as by Brd4-shRNA and depletion of CEBPβ. Inhibitors of Brd4 also dramatically suppress the transcription of Keap1, thereby increasing the expression of anti-oxidant genes through the Nrf2 pathway and reducing the cytotoxicity induced by Brd4 inhibitors. Elimination of ATG3 or KEAP1 expression using CRISPR-cas9 mediated genomic editing markedly reduced autophagy. We conclude that Brd4 plays a significant role in autophagy activation through the direct transcriptional regulation of genes essential for it, as well as through the Keap1-Nrf2 axis in NPMc+ and MLL-fusion AML cells.

## INTRODUCTION

Autophagy is an adaptive survival mechanism that degrades damaged organelles and macromolecules via lysosomes under conditions of cellular stress [1]. While autophagy appears to be critical for the proper maintenance of the metabolism and function of hematopoietic stem cells (HSC) [2–6], its role in the pathogenesis and/or treatment of acute leukemia is controversial and both tumor-promoting and tumor-suppressive functions have been reported [2, 7–9]. It has been shown that conditional knock-out of ATG7 impaired autophagy and induced a pre-leukemic phenotype in HSCs [2], while heterozygous loss of ATG5 enhanced leukemia progression in a mouse model [8]. Conversely, cytoprotective autophagy has been demonstrated in leukemic and other cells subjected to oxidative and metabolic stress [7, 9]. The role of autophagy in acute leukemia appears to vary depending on the leukemia phenotype and genotype.

The mechanisms that result in autophagy activation in leukemic cells are largely unknown. We recently found that autophagy is substantially activated in mutated nucleophosmin (NPMc+) and MLL-fusion AML cells and that inhibitors of the Bromodomain-containing Protein-4 (Brd4) family of proteins markedly reduce autophagy in these subtypes [10]. Brd4 is the best characterized member of the bromo- and extra-terminal (BET) domain family of proteins and has been widely studied in tumor-associated transcriptional programs [11–16]. Brd4-mediated gene-specific targeting is tightly regulated by the cooperative action of a number of other transcription factors including CEBPβ, a hematopoietic transcription factor that physically interacts with Brd4 in a reciprocal manner to regulate the transcription of defined subsets of genes in context-dependent ways [16–20]. CEPBβ also binds to the promoters of a number of autophagy genes [21–23]. We therefore examined the role of Brd4 in regulating a set of genes important in the activation of autophagy that include ATG3, ATG7, and CEBPβ.

It is known that excessive generation of reactive oxygen species (ROS) can activate autophagy [24–27]. Increased ROS production may result from excessive production of ROS through oxidative metabolism and/or a decrease in antioxidant defenses mediated by NF-E2-related factor 2 (Nrf2) [24, 28–32]. Nuclear Nrf2 binds to antioxidant response elements (ARE) within the promoters of genes encoding antioxidant and detoxifying enzymes including NADPH quinone oxidoreductase 1 (NQO1) [33–35]. A previous study has shown that Brd4 suppressed Nrf2-mediated antioxidant gene expression, most likely by enhancing the expression of the Nrf2 ubiquitin ligase Keap1 and thereby increasing ROS levels [36, 37]. However, a specific role for the Keap1/Nrf2 axis in the induction of autophagy in leukemia has not been shown. The present study investigates the relative roles of Brd4 and the Keap1/Nrf2 axis in the regulation of key autophagy-related genes and autophagy in acute leukemic cells.

## RESULTS

### Brd4-dependent activation of autophagy genes and CEBPβ in NPMc+ and MLL-fusion AML

**Figure 1:**
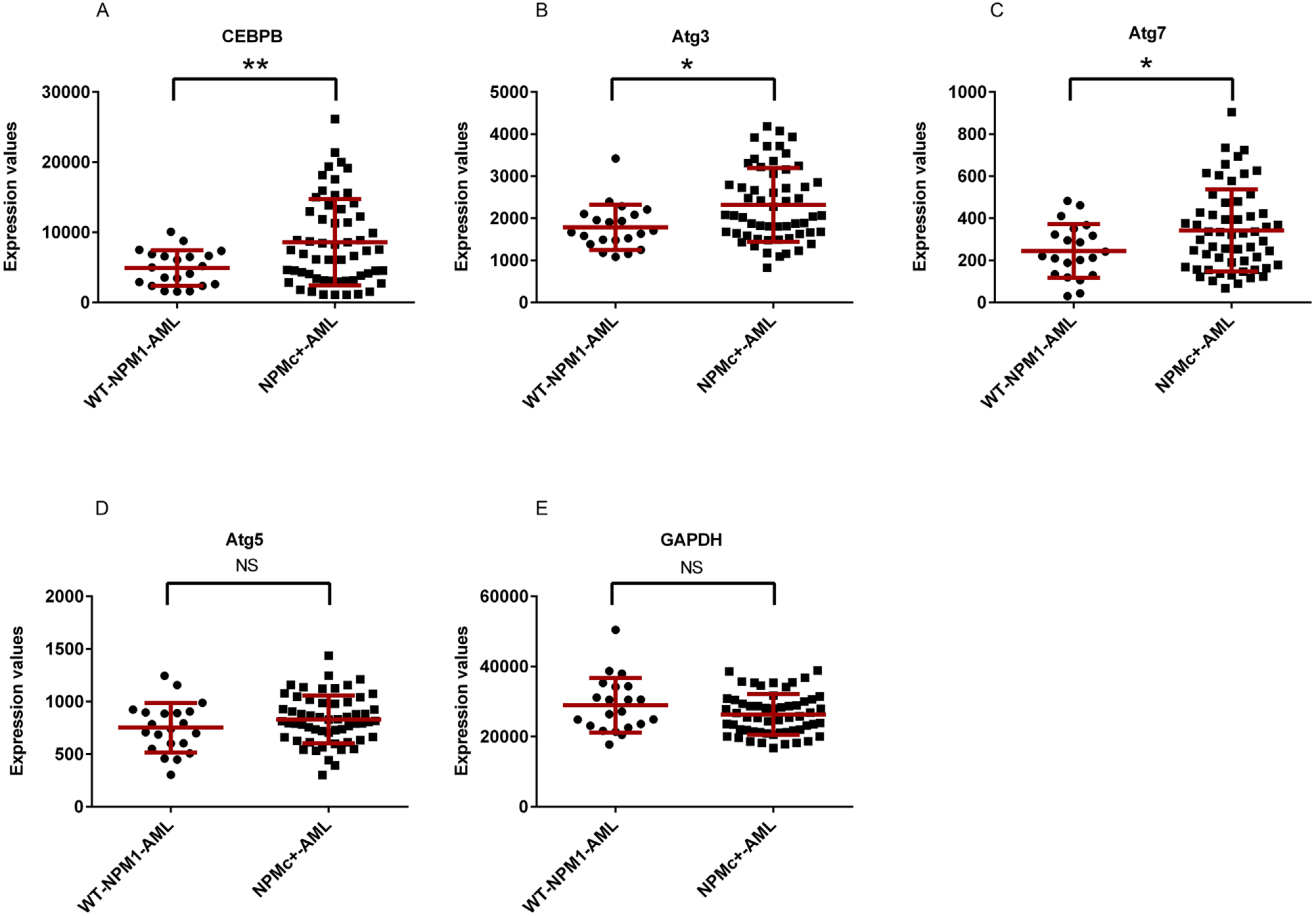
Expression of CEPBβ, Atg3, and Atg7 in NPMc+ AML The relative levels of mRNA expression data in primary AML samples were obtained from the *expression* omnibus (GEO: GDS4500 and GDS4501) [38]. The average mRNA expression levels for CEBPβ, Atg3, and Atg7 **(A, B, C, D, E)** were significantly higher in AML cells expressing NPMc+ than in wt-NPM1. Each symbol represents a value from an individual patient. Data show mean +/− SD. Asterisks (^*^) and (^**^) indicate *p* < 0.05 and *p* < 0.01, respectively.

In order to determine whether a relationship exists between the expression of key autophagy-associated genes and leukemia phenotypes, we analyzed gene expression data extracted from the GEO database for primary AML samples [38]. The mean mRNA expression of Atg3, Atg7, and CEBPβ was significantly increased in NPMc+ AML as compared to non-NPMc+ AML (Figure 1A, 1B and 1C). In contrast, there was no differential expression of Atg5 or GAPDH (Figure 1D and 1E).

**Figure 2:**
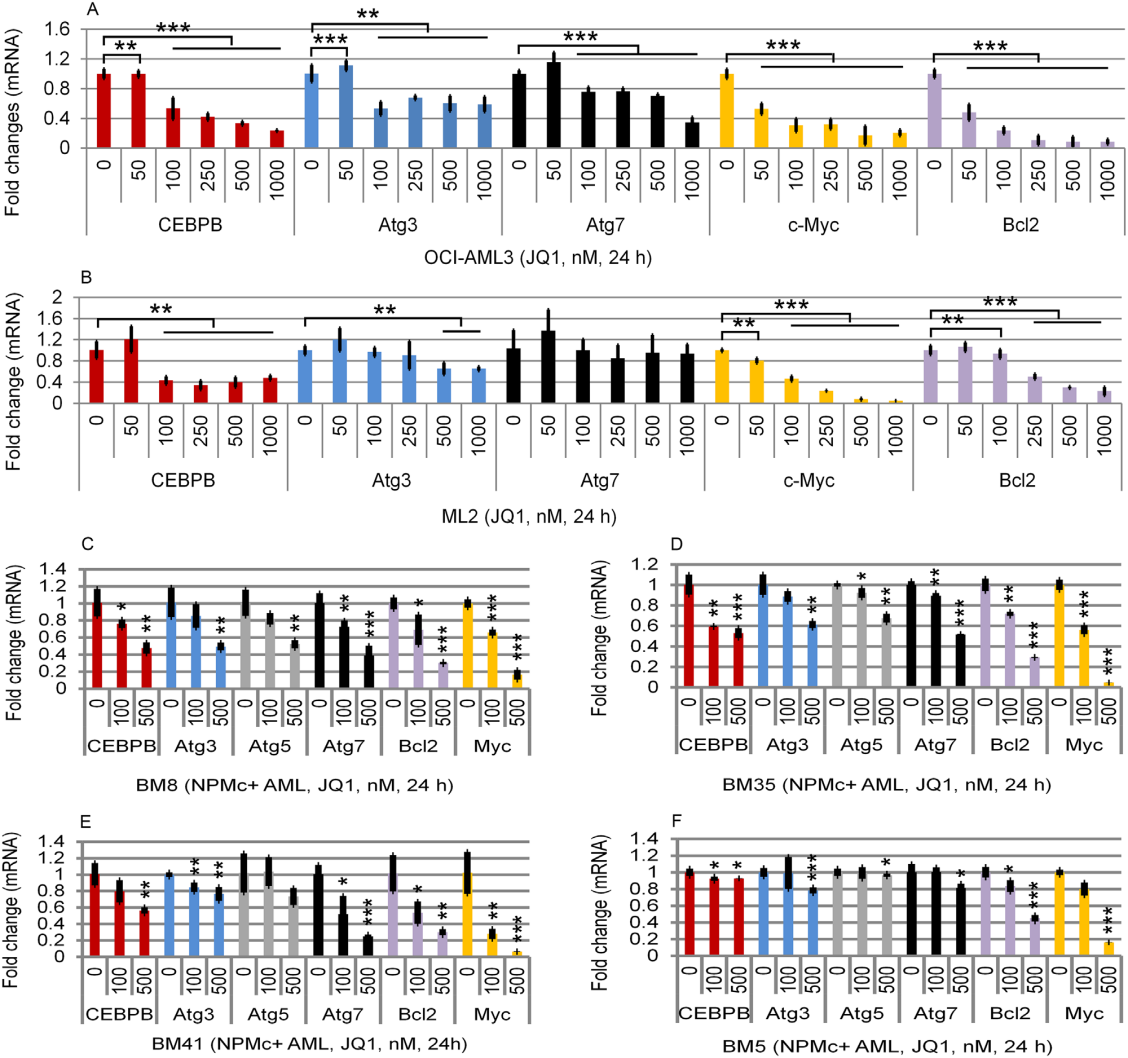
Effects of JQ1 on CEBPβ, Atg3, Atg5, and Atg7 expression in cell lines and in primary AML cells Effects of JQ1 on mRNA levels of CEBPβ, Atg3, Atg7, c-myc, and Bcl2 on OCI-AML3 cells **(A)**, ML2 cells **(B)** and on primary NPMc+-expressing BM8-AML **(C)**, BM35-AML **(D)**, BM41-AML **(E)**, and BM5-AML **(F)** cells. The characteristics of the primary AML cells are shown in Supplementary Table 1. Cells were untreated or treated with JQ1 at the concentrations shown for 24 h, followed by q-PCR analysis. The relative levels of mRNA expression were calculated using the 2^−ΔΔCt^ method after normalization to the GAPDH level and were expressed as fold changes relative to control (set at 1). The mean ± S.D. of four replicates is shown. Asterisks (^*^), (^**^), and (^***^) indicate *p* < 0.05, *p* < 0.01, and *p* ≤0.001 in relation to untreated controls.

As noted earlier, there is evidence linking Brd4 with autophagy in both NPMc+ and MLL-fusion AML [10]. We therefore asked whether Brd4 itself regulates the expression of specific autophagy-associated genes. As shown in Figure 2, the Brd4 inhibitor JQ1 markedly reduced the mRNA expression levels of Atg3, Atg7, and CEBPβ in the OCI-AML3 NPMc+ cell line (Figure 2A), as well as in primary NPMc+ AML (Figure 2C, 2D, 2E, 2F, and Supplementary Table 1), and ML2 MLL cells (Figure 2B). The inhibitory effect of JQ1 on CEBPβ mRNA expression was comparable its effect on c-Myc and Bcl2 mRNAs (Figure 2A-2D), both well established as Brd4 target genes.

**Figure 3:**
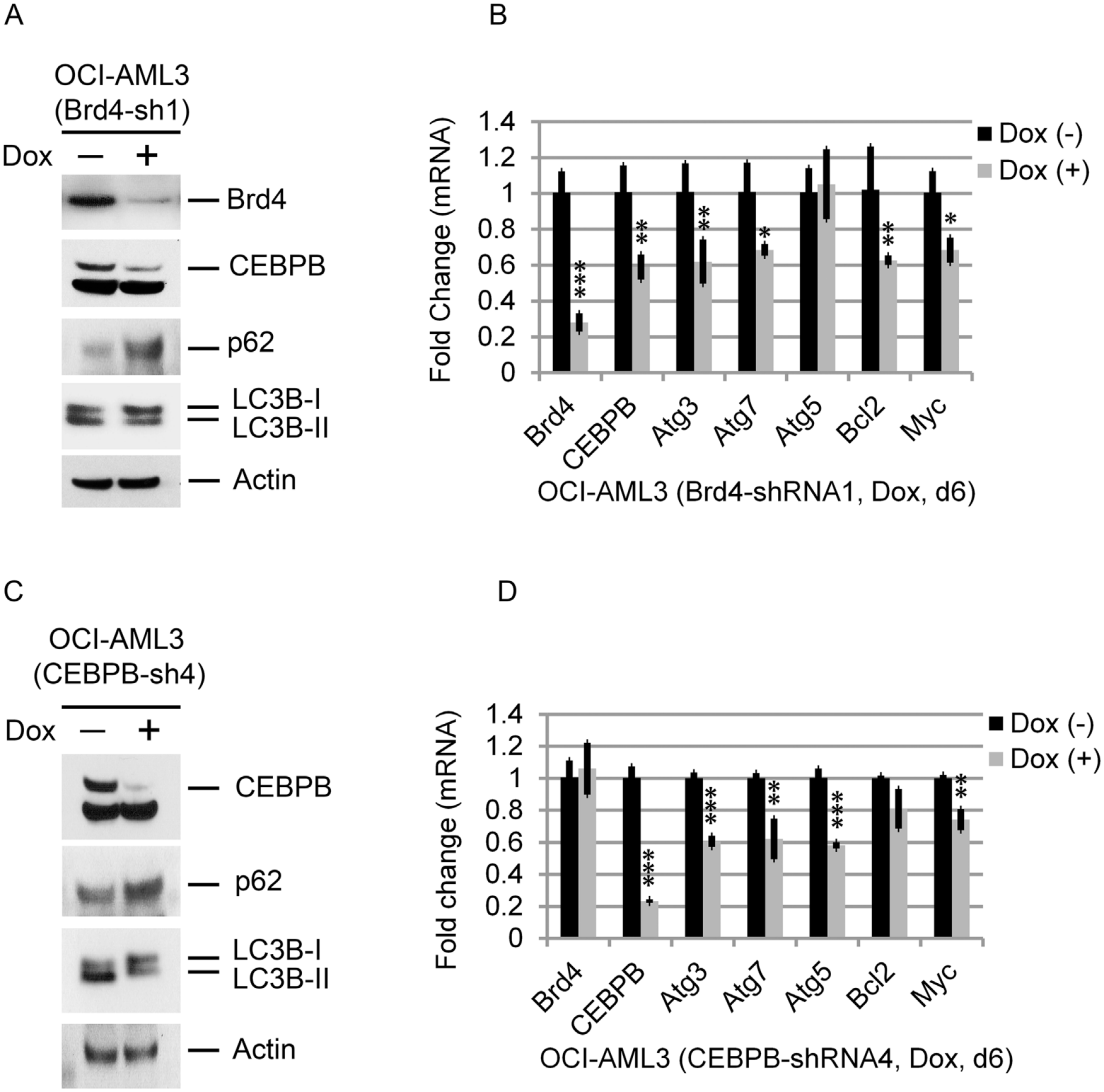
Effects of Brd4 and CEBPβ depletion OCI-AML3 cells were treated with vehicle or doxycycline to express Brd4 or CEBPβ shRNA and effects on gene expression assessed after 6 days. **(A, C)** Effects of Brd4 and CEBPβ depletion on p62, LC3B, and CEBPβ expression. **(B, D)** qPCR analysis of mRNA levels, as indicated. Bar graphs represent the mean ± S.D. of four replicates. Asterisks (^*^), (^**^), and (^***^) indicate *p* < 0.05, *p* < 0.01, and *p* < 0.001, respectively, in relation to cells without doxycycline induction.

To further demonstrate a regulatory role for Brd4 in autophagy, we inducibly depleted Brd4 expression in OCI-AML3 cells with shRNA. Knockdown of Brd4 decreased the expression of CEBPβ protein (Figure 3A) and mRNA (Figure 3B). Similarly, the expression of Atg3 and Atg7 was significantly reduced (Figure 3B). Major reductions in the expression of these and other autophagy-related genes were also seen in murine MLL-AF9-expressing AML RN2 cells following Brd4 depletion (Supplementary Figure 1) [19]. Brd4-shRNA concomitantly reduced the conversion of LCB-I to LC3B-II and decreased the degradation of p62, both well-established markers of autophagy (Figure 3A and 3B). Depletion of CEBPβ using shRNA similarly reduced the expression of Atg3and Atg7 while decreasing the conversion of LC3B-I to LC3B-II and the degradation of p62 (Figure 3C and 3D). These data suggest that the dependence of autophagy on Brd4 may, at least in part, be mediated through its interaction with and/or regulation by CEBPβ in these cell types.

**Figure 4:**
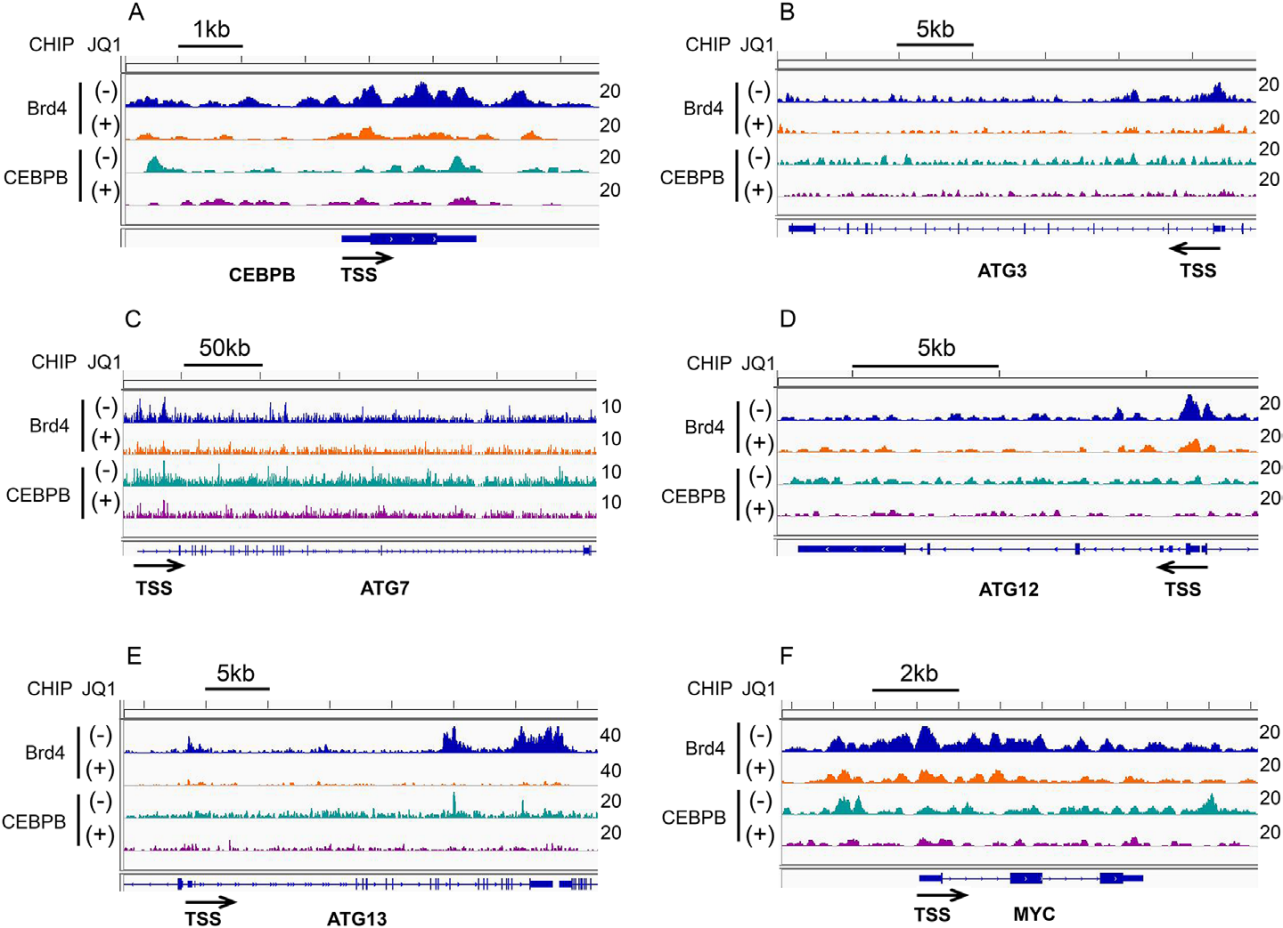
Effects of JQ1 on Brd4 and CEBPβ binding to the promoters or enhancers of autophagy-related genes OCI-AML3cells were treated in the absence or presence of 500 nM JQ1 for 24 h, followed by Brd4- and CEBPβ-ChIP-seq, as described in the Materials and Methods. Genome browser (hg38) views of ChIP-seq peaks at the following loci: CEBPβ **(A)**, ATG3 **(B)**, ATG7 **(C)**, ATG12 **(D)**, ATG13 **(E)**, and c-MYC **(F)**. The location of each gene is shown at the bottom of the panels and the calculated ChIP-seq enrichment values are indicated on the right.

**Figure 5:**
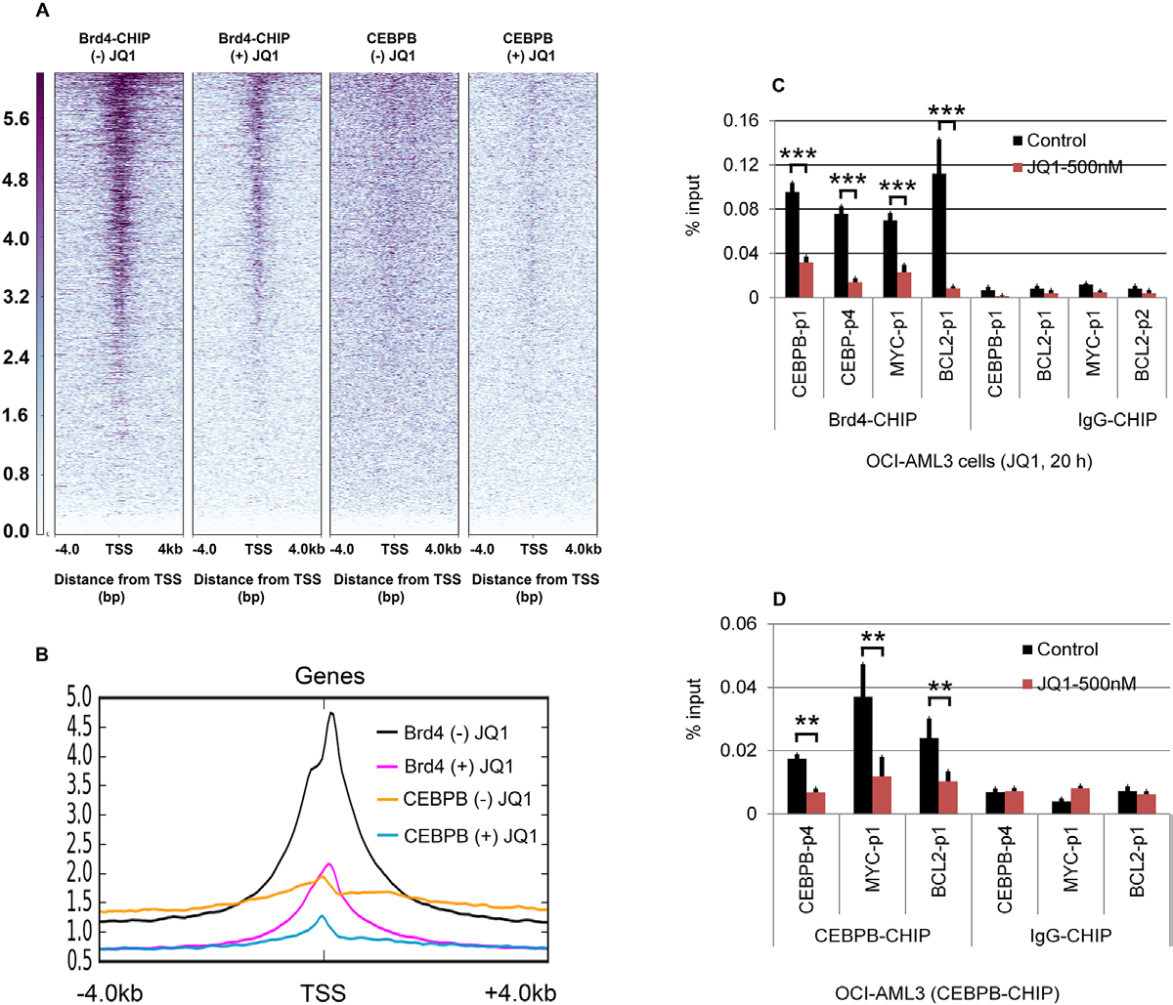
Effect of JQ1 on ChIP-seq density heat maps of Brd4 and CEBPβ binding profiles OCI-AML3 cells were treated with or without 500 nM JQ1 for 24 h, followed by Brd4 and CEBPβ ChIP-seq. **(A)** ChIP-seq density heat maps of Brd4 and CEBPβ-ChIP-seq centered on the transcription start site (TSS) of annotated genes with 4 kb of flanking sequences. Gene promoters are rank-ordered by the level of Brd4 and CEBPβ enrichment at the TSS in the presence or absence of JQ1. **(B)** Average genome-wide occupancies in the presence or absence of JQ1 are shown for Brd4 and CEBPβ; Effect of JQ1 on promoter binding by Brd4 **(C)** and CEBPβ **(D)**, as assessed by ChiP-qPCR. Error bars represent mean +/−SD from four replicates.

**Figure 6:**
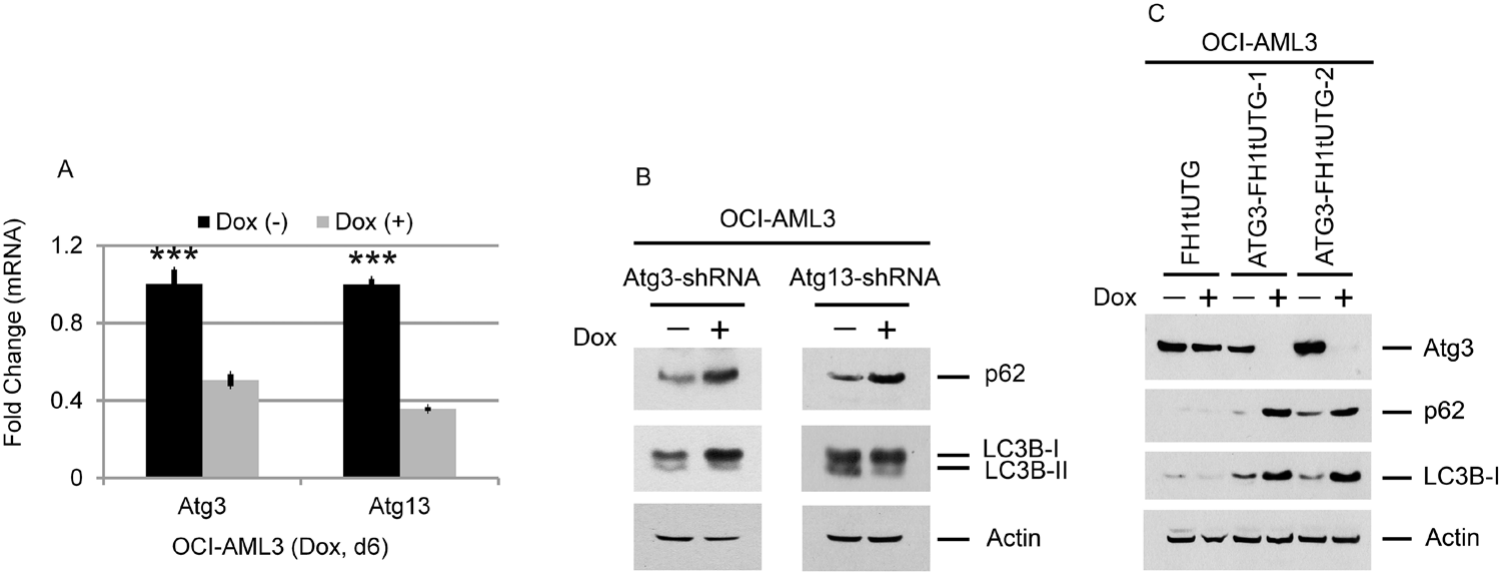
Effects of Atg3 and Atg13 depletion on the autophagy markers p62 and LC3B OCI-AML3 cells stably expressing inducible Atg3- or Atg13-shRNA were treated with or without doxycycline for six days, followed by qPCR analysis **(A)** and Western blotting of the proteins shown **(B)**. Bar graphs represent the mean ± S.D. of four replicates. Asterisks (^***^) indicate *p* < 0.001, in relation to cells without doxycycline induction. **(C)** Effect of ATG3 depletion on p62 and LC3B in OCI-AML3 cells. OCI-AML3 cells stably expressing inducible ATG3 gRNA were treated with vehicle or doxycycline to induce genome editing for six days, followed by Western blotting of the proteins shown.

Using ChIP-seq, we determined that Brd4 is present at the promoters and enhancers of CEBPβ, ATG3 and ATG7, and is reduced at all loci with JQ1 treatment (Figure 4A-4F). In addition, we found robust enrichment of Brd4 at the ATG12 and ATG13 genes and a reduction in both occupancy and mRNA expression following JQ1 treatment (Figure 4D, 4E and Supplementary Figure 2). CEBPβ co-locates with Brd4 at promoter and enhancer regions of CEBPβ itself (Figure 4A), and of ATG7 (Figure 4C), ATG13 (Figure 4E), and c-MYC (Figure 4F), and is similarly reduced by JQ1 treatment. Comparison of the genome-wide occupancies of Brd4 with those of CEBPβ in the absence or presence of JQ1 demonstrates a similar pattern for a wide variety of genes, as shown by heatmaps (Figure 5A) as well as by average binding profiles (Figure 5B). JQ1 treatment also significantly decreased the occupancy by Brd4 (Figure 5C) and CEBPβ (Figure 5D) at both the promoter (CEBPβ-p1) and enhancer (CEBPβ-p4) regions of CEBPβ. Genome browser views of ChIP-seq peaks extracted from the GEO dataset GSE66123 [19] revealed similar enrichment patterns of Brd4 and CEBPβ at the promoters or enhancers of ATG3, ATG7, ATG12, ATG13, and c-MYC in murine MLL-AF9 AML cells, in association with the histone activation marks H3K27ac and H4K8-Ac (Supplementary Figure 3). These results, taken together, support a direct role for Brd4 in association with CEBPβ in the regulation of autophagy. To determine the association betwee Atg3 and Atg13 expression and autophagy, OCI-AML3 cells were subjected to inducible depletion of Atg3 and Atg13 using shRNA. A 2-3-fold reduction in the expression levels of *Atg3 or Atg13* increased levels of both p62 and LC3B-I (Figure 6A, 6B). A more complete knock-out of ATG3 by CRISPR-cas9 mediated genome editing (ATG3 gRNA1 and gRNA2) further increased p62 and LC3B-I protein expression (Figure 6C), reflecting a decrease in autophagy.

### Regulation of the Keap1-Nrf2 axis by Brd4

**Figure 7:**
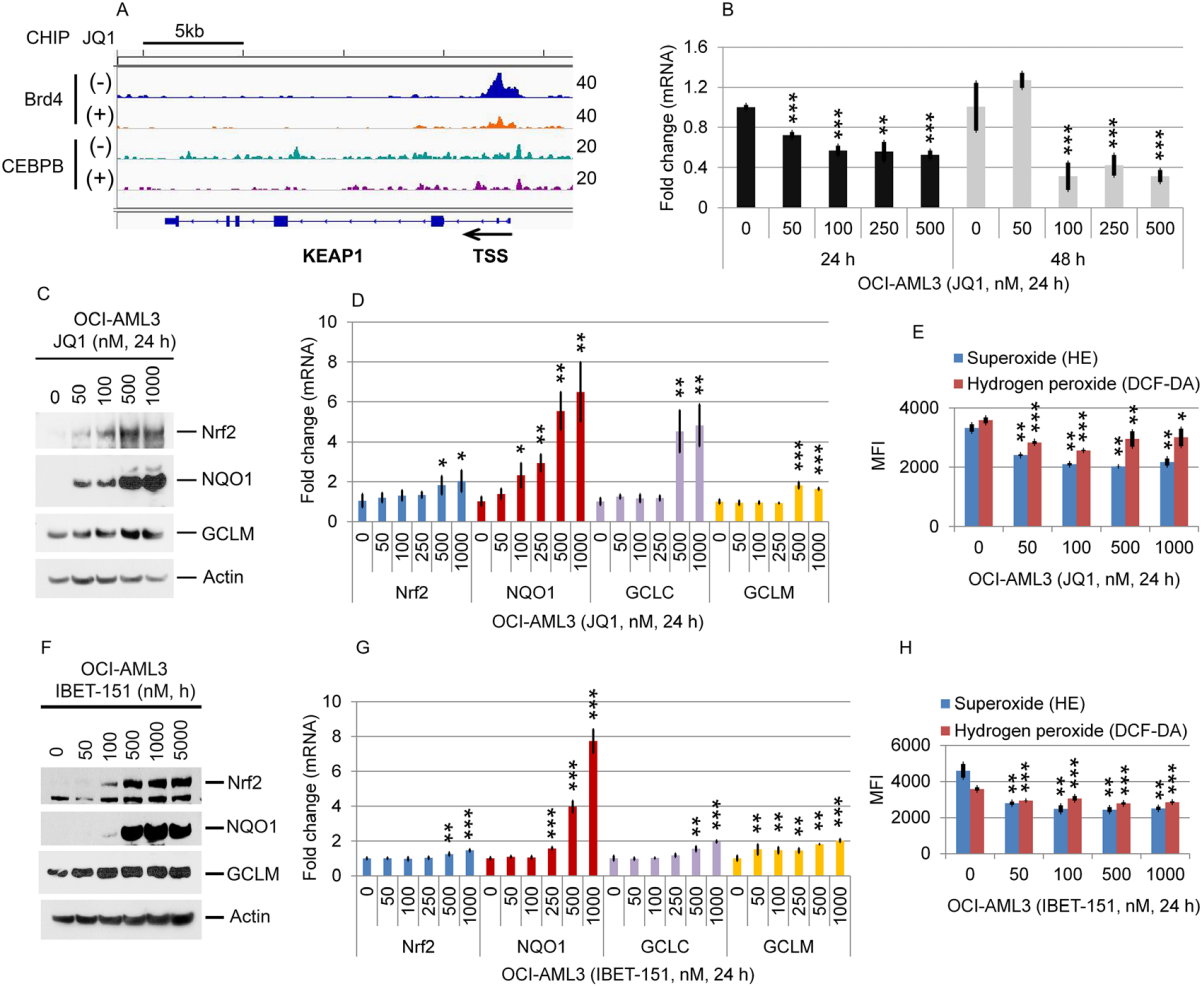
Effects of JQ1 on the Nrf2 antioxidant pathway **(A)** OCI-AML3 cells were cultured in the presence or absence of 500 nM JQ1 for 24 h, followed by Brd4- and CEBPβ-ChIP-seq. The representative genome browser views of Brd4- or CEBPβ-binding peaks adjacent to the Keap1 locus are shown. **(B)** OCI-AML3 cells were treated with indicated concentrations of JQ1 for 24 h and 48 h, followed by q-PCR analysis of Keap1 mRNA expression. **(C, D, E, F, G, H)** OCI-AML3 cells were treated with JQ1 or I-BET-151 at the indicated concentrations for 24 h, followed by Western blotting (C, F), qPCR analysis of Nrf2, GCLC, GCLM, and NQO1 (D, G), and quantitative measurement of superoxide and hydrogen peroxide production (E, H).

**Figure 8:**
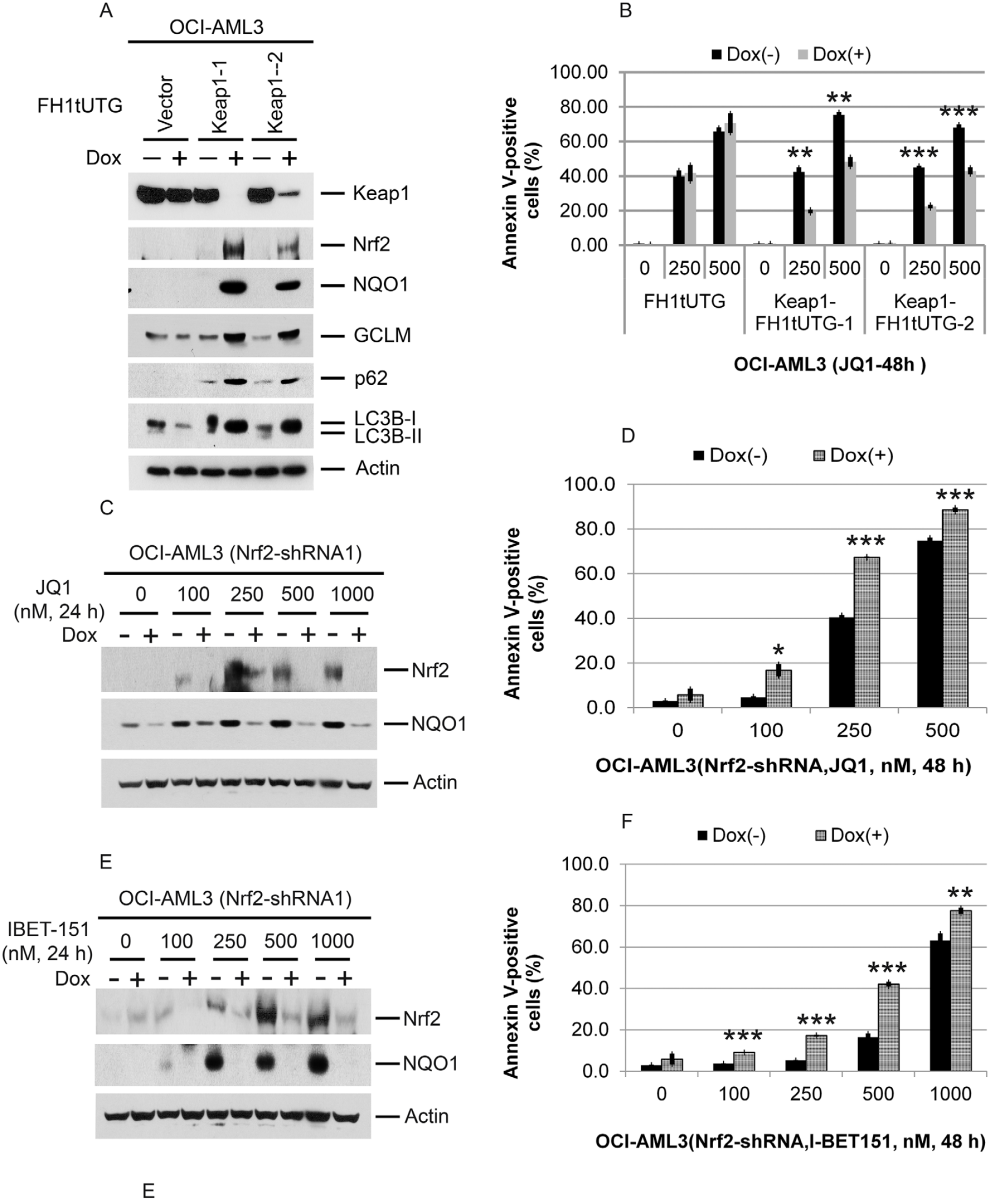
Effects of Keap1 depletion on p62 and LC3B; effects of Keap1 or Nrf2 depletion on Nrf2 NQO1, and apoptosis induced by Brd4 inhibition **(A)** Effect of Keap1 depletion by CRISPR-cas9 mediated genome editing on p62, LC3B, Nrf2, GCLC, GCLM, and NQO1 in OCI-AML3 cells. OCI-AML3 cells stably expressing inducible KEAP1 gRNA were treated with vehicle or doxycycline to induce genome editing of KEAP1 for six days, followed by Western blotting of the proteins shown. **(B)** Effects of CRISPR-cas9 mediated genome editing of KEAP1 on JQ1-induced apoptosis. OCI-AML3 cells stably expressing inducible KEAP1 gRNA were incubated with or without doxycycline for five days, followed by treatment with JQ1 at the indicated concentrations for 48 h and analysis of Annexin V positivity. Bar graphs represent the mean ± S.D. of biological triplicates. Asterisks (^**^) and (^***^) indicate *p* < 0.01 and *p* < 0.001, respectively, in relation to cells without doxycycline induction. **(C, D)** OCI-AML3 cells stably expressing inducible Nrf2 shRNA were incubated with or without doxycycline for five days, followed by treatment with JQ1 and I-BET-151 at the indicated concentrations for 24 h and Western blot analysis of the proteins shown. **(E, F)** OCI-AML3 cells were treated as above, followed by treatment with JQ1 (E) or I-BET-151 (F) at the indicated concentrations for 48 h and analysis of Annexin V positivity. Bar graphs represent the mean ± S.D. of biological triplicates. Asterisks (^**^) and (^***^) indicate *p* < 0.01 and *p* < 0.001, respectively, in relation to control cells without doxycycline induction.

Considerable data support a direct relationship between ROS activation and autophagy [24–27]. In order to determine whether Brd4 or CEBPβ plays an important role in this relationship, we asked whether Brd4 and/or CEBPβ regulate the Keap1/Nrf2 anti-oxidant pathway [33–35]. As shown in Figure 7A, there is an enrichment of Brd4, but not of CEBPβ, at the KEAP1 promoter that decreases following JQ1 treatment. BET inhibition also significantly reduces Keap1 mRNA expression (Figure 7B) while simultaneously increasing Nrf2, NQO1, GCLM, and GCLC protein and/or mRNA levels (Figure 7C, 7F, 7D, 7G) and decreasing superoxide and hydrogen peroxide levels (Figure 7E and 7H). These results support a direct role of Brd4 in regulating the Keap1-Nrf2 antioxidant response and intracellular ROS levels in AML cells. To investigate whether there is a direct relationship between Keap1 expression and autophagy, we knocked out KEAP1 using CRISPR-cas9 genome editing. Loss of Keap1 resulted in a marked increase in the expression of Nrf2 and NQO1 as expected (Figure 8A), as well as an increase in p62 and a reduction in the conversion of LC3B-I to LC3B-II (Figure 8A) reflecting a decrease in autophagy. The loss of Keap1 also reduced the extent of apoptosis induced by either JQ1 or IBET-151 (Figure 8B), while depletion of Nrf2 increased the apoptosis that results from exposure to these BET inhibitors (Figure 8C-8F). These results indicate that the direct regulation of the Nrf2 antioxidant response by Brd4 plays an important role in both autophagy and the cytotoxic response of certain AML cells to Brd4 inhibitors, a finding of potential clinical relevance.

## DISCUSSION

Since the discovery of Brd4 as an epigenetic regulator, a number of studies have characterized target genes and molecular pathways that are regulated by it [11–16, 19]. Recent studies have suggested a model in which Brd4 acts as a scaffold to recruit multiple regulatory proteins such as the hematopoietic transcription factor CEBPβ through direct protein-protein interactions [16, 19, 21–23]. The recent finding that NPMc+ and MLL-fusion AML cell lines and primary leukemic cells have sustained activation of autophagy and that autophagy is reduced with Brd4 inhibition [10] suggested that Brd4 is a regulator of autophagy. The present study provides more direct evidence that Brd4 in conjunction with CEBPβ plays an important role in the transcriptional regulation of autophagy genes in certain AML cells. Inhibition of Brd4 function with both well-validated inhibitors and inducible Brd4 shRNAs reduced the expression of Atg3, Atg7, and CEBPβ, each of which plays an important role in the induction of autophagy. Genome-wide occupancy studies of Brd4 using Brd4-ChIP-seq demonstrated that Brd4 is located at the promoter and enhancer regions of CEBPβ and is present in conjunction with CEBPβ at several other autophagy-associated genes, suggesting an integral role for CEBPβ in autophagy. This result is further supported by the marked reduction of Atg3 and Atg7 expression that follows depletion of CEBPβ. These data, together with those obtained by Roe et al [19], indicate that Brd4 acts as an upstream regulator responsible for the recruitment of CEBPβ to core genes enabling autophagy in select cell types.

Somatic mutations in KEAP1 and NRF2 occur in a variety of cancer types and are important mediators of aberrant anti-oxidant responses [39, 40]; however, no such mutations have been reported to date in human leukemia cells (The Cancer Genome Atlas, TCGA, database) [41–43]. Our study supports a model in which Brd4 increases the expression of Keap1, resulting in down-regulation of the Nrf2-antioxidant pathway and increases in both ROS levels and autophagy in NPMc+ AML cells [36, 37]. Conversely, a reduction in Keap1 expression by Brd4 inhibition or CRISPR-cas 9 excision increases the Nrf2 anti-oxidant response, reduces ROS levels, and suppresses autophagy. Although NPMc+ AML and MLL-fusion AML cells have distinct mutational profiles, these two subtypes share a number of similarities including aberrant HOX gene expression [38, 44–46], activation of the core Brd4 transcriptional program [10, 47, 48], and sensitivity to BET inhibitors *in vitro* [10, 47, 48]. These similarities led us to compare the effects of Brd4 on autophagy, first noted in NPMc+ AML, in both subtypes of AML. Our present study shows that NPMc+ and MLL-fusion AML cells also share increased autophagy activity through Brd4 activation. Brd4 appears to activate autophagy directly through modulating the expression of autophagy-associated genes and indirectly through increasing ROS by KEAP1 activation and the subequent downregulation of the Nrf2 antioxidant pathway.

Brd4 is a validated drug target in leukemia and Brd4 chromatin occupancy in AML correlates with the transcriptional activation of a number of essential hematopoietic transcription factors [19, 49]. Several BET inhibitors are in development and have been shown to have activity across various subtypes of AML in mouse models including MLL-fusion leukemias [48], as well as leukemias bearing mutations in NPM1, Flt3-ITD and DNMT3A [47, 50, 51]. It is apparent from our data that the expression of a subset of autophagy genes and of Keap1 are reduced by Brd4 inhibitors and that the effect of Keap1 reduction counterbalances the apoptosis induced by JQ1. As these inhibitors enter the clinic, there will be the opportunity for a prospective examination of their role in regulating autophagy and the Keap1-Nrf2 axis in relation to their therapeutic efficacy in primary leukemic cells.

## MATERIALS AND METHODS

### Culture of primary AML cells and cell lines

Ficoll-purified mononuclear cells from the bone marrow or peripheral blood of AML patients were obtained after informed consent according to institutional guidelines (Stanford University Institutional Review Board No. 6453). Ficoll-purified mononuclear cells were cultured in modified culture medium consisting of equal parts of EGM-2 complete medium (Lonza, Cologne) and SFEM complete medium (Stem Cell Technology). The ML-2 and 293 T cell lines were obtained from American Type Culture Collection and the OCI-AML3 cell line from the German Collection of Microorganisms and Cell Cultures. 293T cells were cultured in Dulbecco’s modified Eagle medium (DMEM) supplemented with 10% heat-inactivated fetal bovine serum (FBS), 2 mM glutamine, 100 U ml^−1^ penicillin and 100 μg/ml streptomycin. The OCI-AML3 cell line was maintained in MEM-alpha medium supplemented with 20% heat-inactivated fetal bovine serum (FBS), 2 mM glutamine, 100 U ml^−1^ penicillin and 100 μg/ml streptomycin. All experiments were initiated at a cell density of 1×10^5^ to 4×10^5^ cells/ml.

### RNA isolation and Real-time RT-PCR analysis

RNA extraction and q-PCR were performed as described previously [10]. The specific primers used are listed in the Supplementary Table 2.

### Lentiviral vector constructs and establishment of stable cell lines

The doxycycline-inducible Brd4, CEBPβ, Atg3, Atg13, and Nrf2-shRNA lentiviral constructs were purchased from Open Biosystems (Huntsville, AL, USA). The information for clone IDs and the mature antisense sequences were provided in Supplementary Table 3. OCI-AML3 cells were infected with individual shRNAs as described [10], sorted for YFP fluorescent after overnight treatment with doxycycline, and cultured in doxycycline-free medium for three weeks.

### Western blot

Cell lysis and western blot were performed as described previously (Huang M, Leukemia 2013). Antibodies used for Immunoblots: anti-LC3B ( #2775, Cell Signaling), anti-Keap1 (sc-365626, G-2, Santa Cruz Biotechnology), anti-Atg3 (sc-393660, A-3, Santa Cruz Biotechnology), anti-NQO1 (sc-393736, F-8, Santa Cruz Biotechnology), anti-GCLM (Ab124827, Abcam), anti-Bcl2 (sc-56015, 100/D5, Santa Cruz Biotechnology), anti- p62 (sc-25575, H-290, Santa Cruz Biotechnology), anti-actin (A5441, Sigma), and anti-Nrf2 (sc-13032, H-300, Santa Cruz Biotechnology), anti-Brd4 (A301-985A50, BETHYL Laboratories). Anti-CEBPβ (sc-7962, H-7, Santa Cruz Biotechnology).

### Flow cytometric analyses

Flow cytometric analysis was performed as described previously (Huang M, Cancer research, 2009).

### Generation of CRISPR-cas9 edited cell lines

Genome-editing was performed as described previously (Aubrey, B. J. et al. Cell Rep. 10, 1422–1432, 2015) through the sequential generation of mCherry-Cas9 and inducible gRNA expressing cells. OCI-AML3 cell lines stably expressing mCherry-Cas9 were generated. After sorting of Cas9 expressing mCherry positive cells by FACS, the target specific gRNAs were cloned into the FH1tUTG lentiviral vector and introduced into Cas9 expressing OCI-AML3 cells, followed by FACS sorting of GFP-positive cells stably expressing inducible target-specific gRNAs. The targeting sequences of individual gRNAs used are provided in Supplementary Table 3.

### ChIP–qPCR and ChIP-seq

Chromatin immunoprecipitation (ChIP) was performed as described previously [52]. Briefly, approximately 50 million cells per IP were collected and crosslinked with formaldehyde. Antibodies for Brd4 and CEBPβ (10 ug) were conjugated to 10 μl of Dynabeads bound to protein G (Invitrogen) for overnight at 4°C. Normal rabbit and mouse IgG were used as controls. Antibody-conjugated beads were incubated with chromatin preparations overnight at 4°C. Immunoprecipitated chromatin was washed, treated with proteinase K, and then reverse cross-linked overnight at 65°C. Recovered DNA was extracted by using phenol-chloroform and precipitated with ethanol and glycogen. Input DNA for individual samples was prepared from 5% chromatin before precipitation. Immunoprecipitated DNA and input DNA were amplified with gene-specific primers by qPCR using SYBR-Green. Primer sequences used for the ChIP-assays are listed in the Supplementary Table 4 [53]. Data were calculated as a percentage of input. For ChIP-qPCR three biological replicates were performed per experiment. ChIP-seq libraries were prepared according to the Illumina protocol, and sequenced using a HiSeq (Illumina) by the Stanford Functional Genomics Facility. For Brd4 and CEBPβ ChIP, 75bp paired-end reads were obtained, yielding a minimum of 21 million reads for each sample. ChIP-seq analysis was performed using the public Galaxy online platform (http://www.usegalaxy.org). Sequencing reads were mapped to human reference genome (hg38) using Bowtie aligner with default parameters and peaks were called by MACS version 2.1.0. Peaks were defined as significant with a q value cut-off of 0.05. ChIP-input samples with or without JQ1 treatment were used to construct a peak matrix and to assess or subtract background or non-specific peaks during peak calling. ChIP-seq density heat map and genome-wide occupancy histogram plots were generated using Galaxy. ChIP-seq data have been deposited at Gene Expression Omnibus (GEO; http://www.ncbi.nlm.nih.gov/geo) accession number GSE104745.

### Statistical analysis

Results are expressed as mean value ± S.D. Significance levels were determined using the Student’s *t*-test. ^*^ p < 0.05; ^**^ p < 0.01; ^***^ p < 0.001.

## SUPPLEMENTARY MATERIALS FIGURES AND TABLES



## Abbreviations

HSC: Hematopoietic stem cells
NPMc+: mutated nucleophosmin
Brd4: Bromodomain-containing Protein-4
BET: bromo- and extra-terminal
ROS: reactive oxygen species
Nrf2: NE-E2-related factor 2
ARE: antioxidant response elements
NQO1: NADPH quinone oxidoreductase 1
TCGA: The Cancer Genome Atlas
