# Supplementary material for: Brd4 regulates the expression of essential autophagy genes and Keap1 in AML cells
Source: Oncotarget. 2018 Feb 7;9(14):11665–76. doi: 10.18632/oncotarget.24432 (PMC5837743; doi:10.18632/oncotarget.24432)
Supplement: Supplementary file 1 [file oncotarget-09-11665-s001.pdf]

Brd4 regulates the expression of essential autophagy genes and Keap1 in AML cells

SUPPLEMENTARY MATERIALS

Gene	shRluc	shBrd4
Brd4	3.7	1.8
Atg3	10.1	2.9
Atg4a	2.3	0.8
Atg4b	1.9	0.8
Atg4c	1.2	0.5
Atg5	2.7	2.3
Atg7	1.2	0.3
Atg10	2.7	2.3
Atg12	8.9	2.7
Atg13	2.7	0.5
Atg14	0.6	0.1
Atg16l1	1.5	1.6
Sqstm1	7.7	3.2
Map1lc3b	8.4	3.1
Gabarapl1	2.4	1.1
Becn1	8.2	1.8
Bnip3	10.9	2.1
Cebpb	3.1	1.1
Keap1	0.9	0.2

Supplementary Figure 1: Effects of Brd4 shRNA on the mRNA expression of genes involved in autophagy regulation. Data were extracted from microarray analysis of murine leukemia cells expressing MLL-AF9 fusion with or without Brd4 shRNA [19].

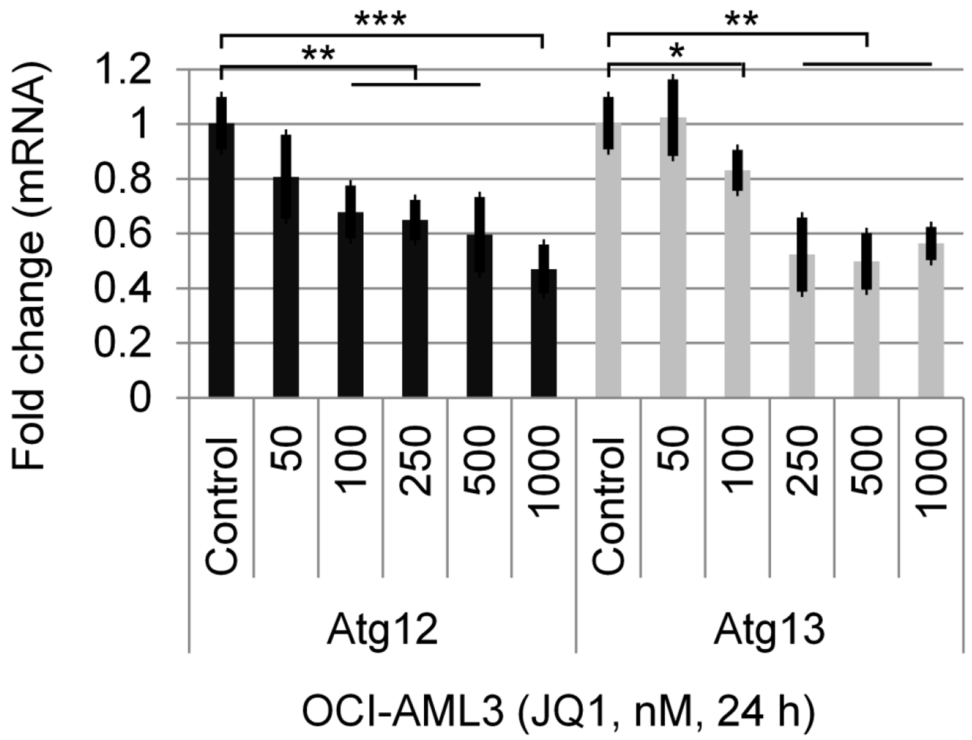

Supplementary Figure 2: Effects of JQ1 on the expression of Atg12 and Atg13. OCI-AML3 cells were untreated or treated with JQ1 at the concentrations shown for 24 h and the expression of Atg12 and Atg13 mRNA in was analyzed by q-PCR. The relative levels of mRNA expression were calculated using the $2^{-\Delta\Delta Ct}$ method after normalization to the GAPDH level and were expressed as fold changes relative to control (set at 1). The mean ± S.D. of four replicates is shown. Asterisks (*), (**), and (***) indicate $p < 0.05$, p<0.01, and $p < 0.001$, respectively, in relation to controls.

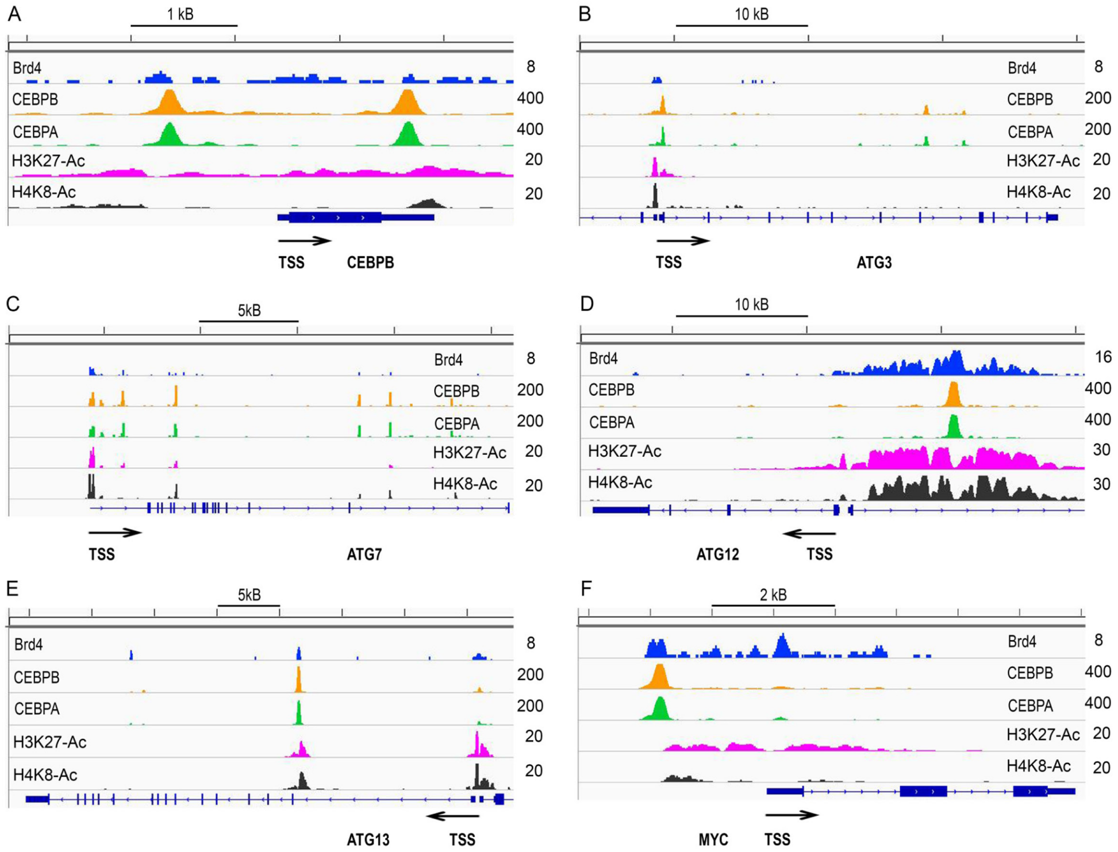

Supplementary Figure 3: Co-localization of Brd4, CEBPβ, CEBPα, and acetylated histone marks on the promoters or enhancers of autophagy-related genes in murine MLL-AF9 AML cells. The representative genome browser views of ChIP-seq peaks for the indicated proteins and histone marks (H3K27-Ac and H4-H8-Ac) at CEBPβ **(A)**, ATG3 **(B)**, ATG7 **(C)**, ATG12 **(D)**, ATG13 **(E)**, and c-MYC **(F)** loci in murine MLL-AF9 cells was extracted form GEO dataset GSE66123 [19]. The relative positions of genes are shown at the bottom, and the calculated ChIP-seq enrichment values at the corresponding gene loci are shown on the right.

Supplementary Table 1: Primary NPMc+ AML profiles in figure 2

AML Patient ID	Age	Disease Status	Karyotype	NPM1 status	FLT3 mutation status	IDH1/2 mutation status	DNMT3A mutation status
BM5	26	Relapsed	NK	NPMc+	WT	No	No
BM8*	68	Relapsed	NK	NPMc+	FLT3-ITD	No	DNMT3A mut (p.Arg882His)
BM35	48	Relapsed	NK	NPMc+	FLT3-ITD	IDH1mut	DNMT3A mut
BM41*	63	Relapsed	NK	NPMc+	FLT3-D835	No	No

Supplementary Table 2: Primer sequences used in real-time RT-PCR analysis

Gene	Primer	Primer sequences	Product size (bp)	Genebank Accession #
NQO1	Forward	5’–GGCAGAAGAGCACTGATCGTA-3’	140	BC007659.2
	Reverse	5’-TGATGG GATTGAAGTTCATGGC-3’		
				S74017.1
Nrf2	Forward	5’-GAGAGCCCAGTCTTCATTGC-3’	203	
	Reverse	5’-TTGGCTTCTGGACTTGGAAC-3’		
GCLM	Forward	5’-GGCACAGGTAAAACCAAATAGTAAC-3’	88	KJ891251
	Reverse	5’-CAAATTGTTTAGCAAATGCAGTCA-3’		
GCLC	Forward	5’-TTGAGGCCAACATGCGAAA-3’	100	NM_001498.3
	Reverse	5’-AGGACAGCCTAATCTGGGAAATG-3’		
GAPDH	Forward	5’-CCCCTTCATTGACCTCAACTACAT-3’	135	NM_002046
	Reverse	5’-CGCTCCTGGAAGATGGTGA-3’		
	Forward	5’-CTGGCGGTGAAGATGCTATT-3’		
	Reverse	5’-GTGGCAGATGAGGGTGATTT-3’		
			201	NM_022488.4
Atg3	Forward	5’-GACGTTGGTAACTGACAAAGTGAAAAAGCA-3’		
	Reverse	5’-CCAAGGAAGAGCTGAACTTGATGCAAG-3’		
Atg5	Forward	5’-ACCCAGAAGAAGCTGAACGA-3’		
	Reverse	5’-CTCATTTGCTGCTTGTTCCA-3’		
Atg7	Forward	5’-TCATCTTCTCCAACGGGTTC-3’	243	NM_006395.2
	Reverse	5’-CCATGGAATCTCCCTCAGAA-3’		
Atg13	Forward	5’-AAAGAAGCGCTTGGAAAACA-3’	160	NM_001142673.2
	Reverse	5’-TGTTTCTTTCCTCCCACGTC-3’		
Brd4	Forward	5’-CTTTGAGTTCGGTGGGGTCA-3’	229	AF386649.1
	Reverse	5’-GGGCCGTACAGTTCCACAAA-3’		
Bcl2	Forward	5’-GCACAGCGACGAGTACAAGA-3’	162	NM_000633.2
	Reverse	5’-AGCTGCTCCACCTTCTTCTG-3’		
CEBPB	Forward	5’-CTTCTCTCCGTCCTCGGATTCT-3’	151	NM_001285879
	Reverse	5’-GAAGGTGATCCAGACTCTGACCTT-3’		
Myc			203	NM_002467.4

Supplementary Table 3: Sequences of mature antisense and gRNA used for knockdown studies

Name	Clone ID	Mature antisense sequence (5' to 3')<br>(in TRIPZ lentiviral vector)
Brd4-shRNA	V3THS_326484	AACAATTTGTAAACATAGT
CEBPB-shRNA	V3THS_371449	GCTTGAACAAGTTCCGCAG
NFE2L2-shRNA	V3THS_306092	AGCATGCTGAAAACTTCGA
Atg3	V2THS_14033	TGCATAAACATATAAGTCC
Atg13	V2THS_72753	TAGCAAGAAGGGACTTCAG
**Name**	**gRNA target sequence (5' to 3')<br>(in FH1tUTG lentiviral vector)**	
ATG3-gRNA1	TTACCTTGAGGACCGGGGTC	
ATG3-gRNA2	AGGTGTAATTACCCCAGAAG	
KEAP1-gRNA1	AGCGTGCCCCGTAACCGCAT	
KEAP1-gRNA2	GCCAATCTGCTCAGCGAAGT	

Supplementary Table 4: Primer sequences for ChIP-qPCR

Name	Forward primer	Reverse Primer
CEBPB-p1 (promoter)	5’-CGCTTAAGTCCCTCCCTAGC-3’	5’-CCAAATGAAATGCCCTCATC-3’
CEBPB-p4 (between last exon and 3’UTR)	5’-TGCAGAAGAAGGTGGAGCAG-3’	5’-TAGCAGTGGCCGGAGGAG-3’
MYC-p1[50] (promoter)	5’- GGACCCGCTTCTCTGAAAGG-3	5’-GCAAGTGGACTTCGGTGCTTACC-3’
BCL2-p1 (promoter)	5’- GATGGGATCGTTGCCTTATG-3’	5’- GCGGAACACTTGATTCTGGT-3’
